# Supplementary material for: Layered liquid crystal elastomer actuators
Source: Nat Commun. 2018 Jun 28;9:2531. doi: 10.1038/s41467-018-04911-4 (PMC6023890; doi:10.1038/s41467-018-04911-4)
Supplement: Supplementary file 1 — Supplementary Information [file 41467_2018_4911_MOESM1_ESM.pdf]

# Layered Liquid Crystal Elastomer Actuators

Tyler Guin[1,2], Michael J. Settle[3,4], Benjamin A. Kowalski[1,2], Anesia D. Auguste[1], Richard V. Beblo[3,4], Gregory W. Reich[3], Timothy J. White[1*]

1 – Air Force Research Laboratory, Materials and Manufacturing Directorate, Wright-Patterson Air Force Base, OH 45433 (USA)

2 – Azimuth Corporation, 4027 Colonel Glenn Hwy, Beavercreek, OH 45431 (USA)

3 – Air Force Research Laboratory, Aerospace Systems Directorate, Wright-Patterson Air Force Base, OH 45433 (USA)

4 – University of Dayton Research Institute, 1700 S Patterson Blvd, Dayton, OH 45469 (USA)

* Correspondence and request for materials should be addressed to T.J.W. (Email: timothy.white.24@us.af.mil)

**Supplementary Figures.**

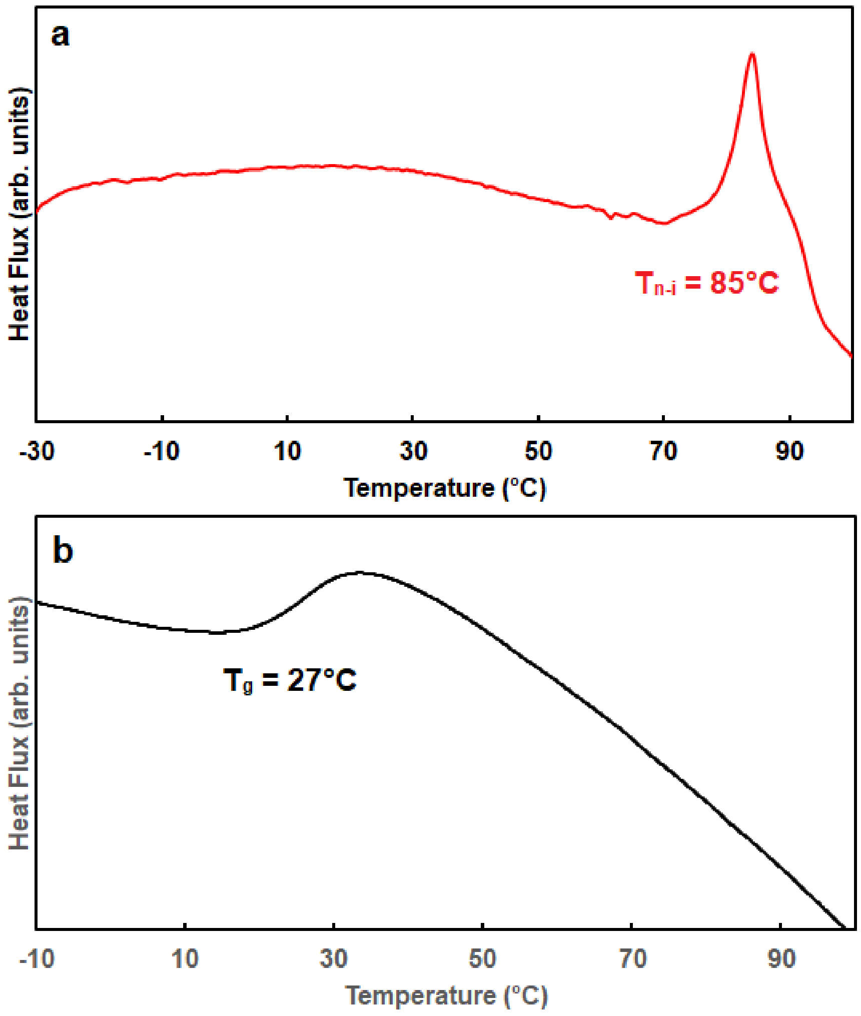


**Supplementary Figure 1.** Differential scanning calorimetry traces of (a) the monomer mixture (pre-polymerization) and (b) the crosslinked LCE film.

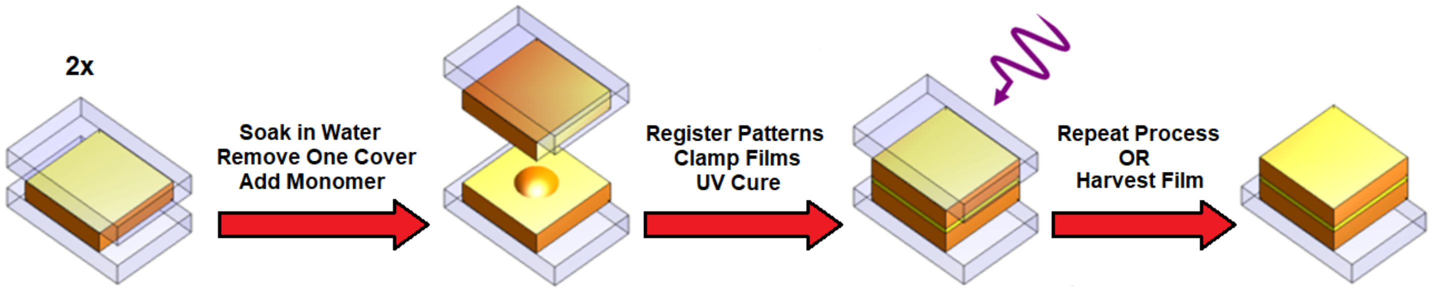


**Supplementary Figure 2.** Illustration of process to prepare laminated LCE actuators.

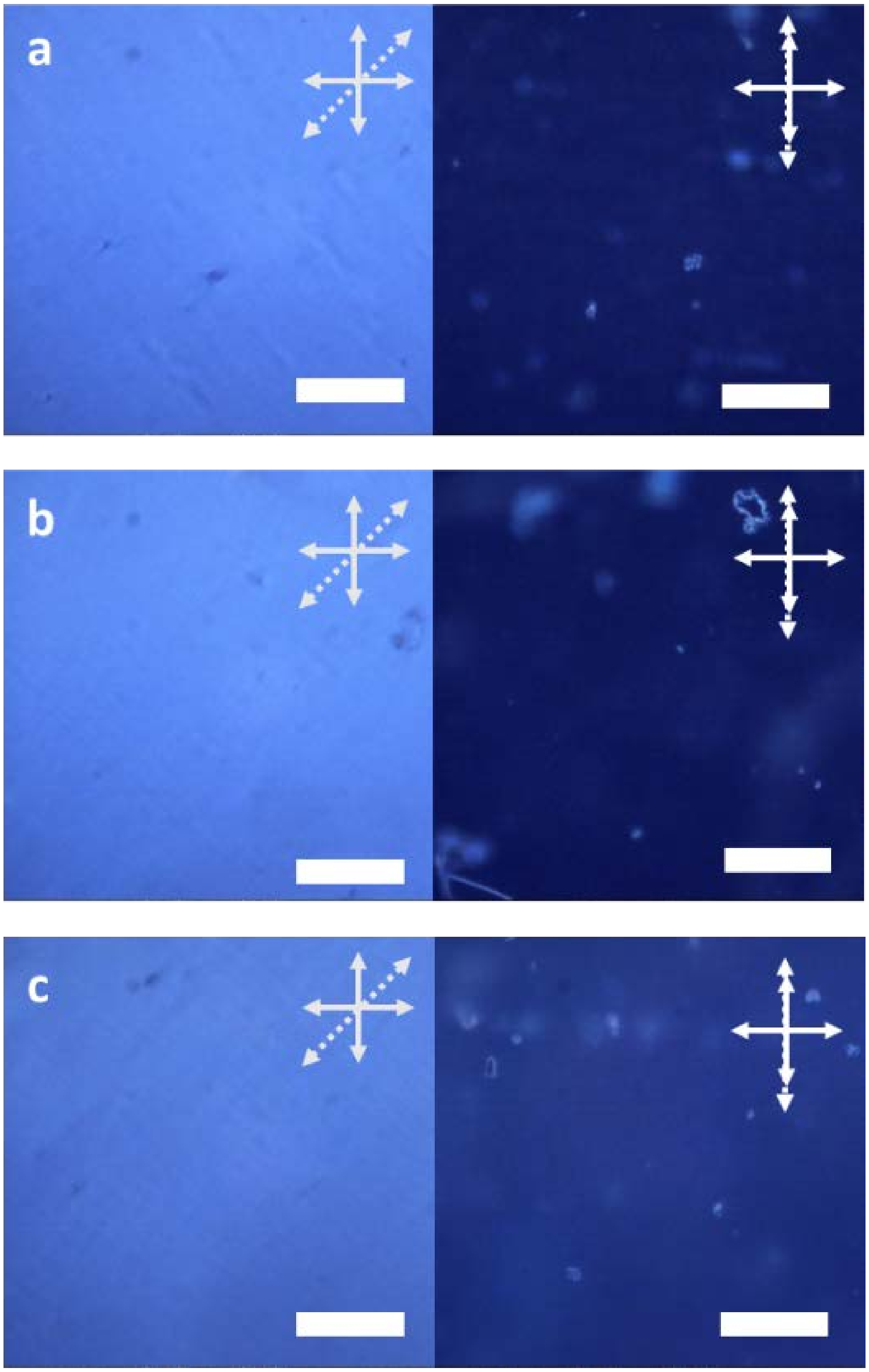


**Supplementary Figure 3.** Polarized optical micrographs of LCE films with (a) one layer, (b) two laminated layers, and (c) four laminated layers. The director is spatially uniform, with orientation shown by dashed arrow. Scale bars 25 µm.

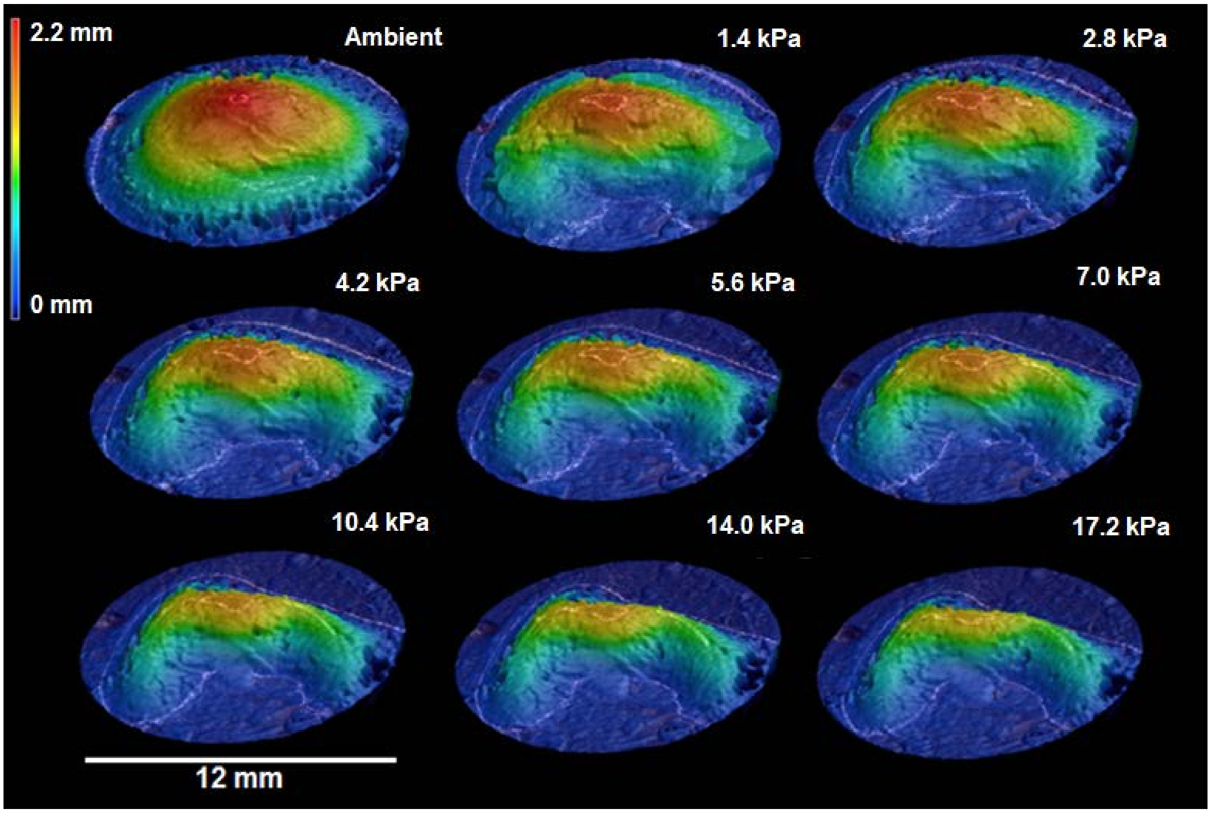


**Supplementary Figure 4**. Deformation of a four-layered LCE laminate, patterned with a single +1 topological defect, as a function of positive air pressure.

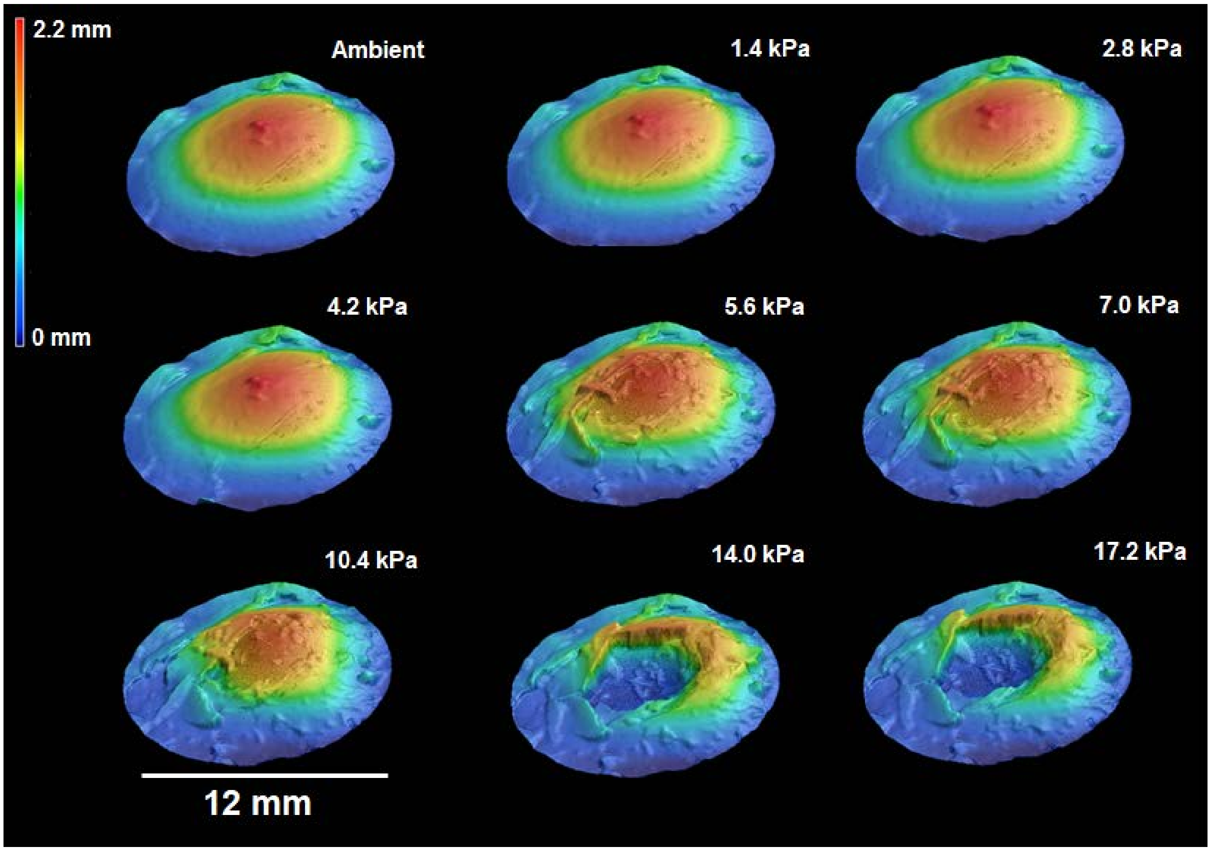


**Supplementary Figure 5**. Deformation of a six-layered LCE laminate, patterned with a single +1 topological defect, as a function of positive air pressure.

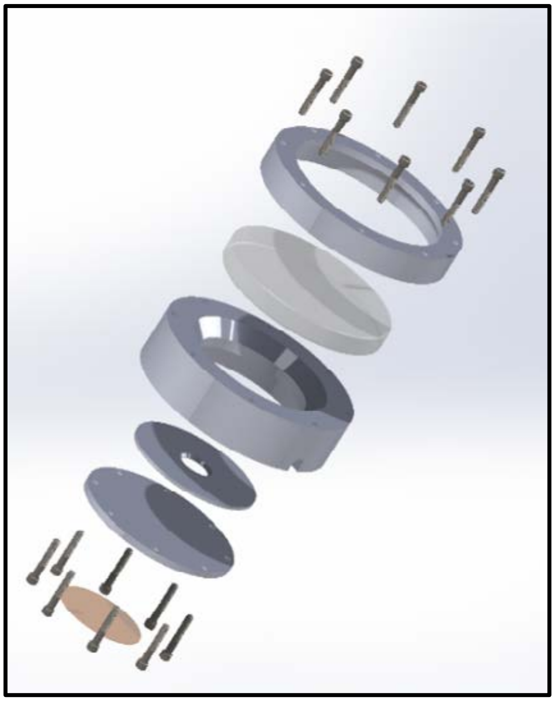

**Supplementary Figure 6**. Exploded diagram of the pressure fixture employed to monitor shape change as a function of pressure.
