# Supplementary material for: Layered liquid crystal elastomer actuators
Source: Nat Commun. 2018 Jun 28;9:2531. doi: 10.1038/s41467-018-04911-4 (PMC6023890; doi:10.1038/s41467-018-04911-4)
Supplement: Supplementary file 3 — Description of Additional Supplementary Files [file 41467_2018_4911_MOESM3_ESM.pdf]

Description of Additional Supplementary Files

File Name: Supplementary Movie 1

Description: A four layer LCE laminate composed of a 2x2 array of +1 topological defects lifts 422x its own weight. Video is sped up by 2.5 times.
